# Supplementary material for: Targeted Immuno-Antiretroviral to Promote Dual Protection against HIV: A Proof-of-Concept Study
Source: Nanomaterials (Basel). 2022 Jun 6;12(11):1942. doi: 10.3390/nano12111942 (PMC9183115; doi:10.3390/nano12111942)
Supplement: Supplementary file 1 [file nanomaterials-12-01942-s001.zip › nanomaterials-1715616-supplementary.pdf]

nanomaterials

Supplementary Materials

Targeted Immuno-Antiretroviral to Promote Dual Protection against HIV: A Proof-of-Concept Study

Subhra Mandal [1,*,†], Shawnalyn W. Sunagawa [1], Pavan Kumar Prathipati [1], Michael Belshan [2], Annemarie Shibata [3] and Christopher J. Destache [1,4]

[1] School of Pharmacy & Health Professions, Creighton University, Omaha, NE 68178, USA; shawnalynsunagawa@creighton.edu (S.W.S.); pavankumarprathipati@creighton.edu (P.K.P.); chrisdestache@creighton.edu (C.J.D.)
[2] Department of Medical Microbiology & Immunology, Creighton University School of Medicine, Creighton University, Omaha, NE 68178, USA; michaelbelshan@creighton.edu
[3] Department of Biology, College of Arts and Sciences, Creighton University, Omaha, NE 68178, USA; annemarieshibata@creighton.edu
[4] School of Medicine, Division of Infectious Diseases, Creighton University, Omaha, NE 68178, USA
* Correspondence: subhra.mandal@unl.edu; Tel.: +1-402-472-5922
† Current address: Center for Virology, School of Biological Sciences, University of Nebraska-Lincoln, Lincoln, NE 68583, Nebraska, USA.

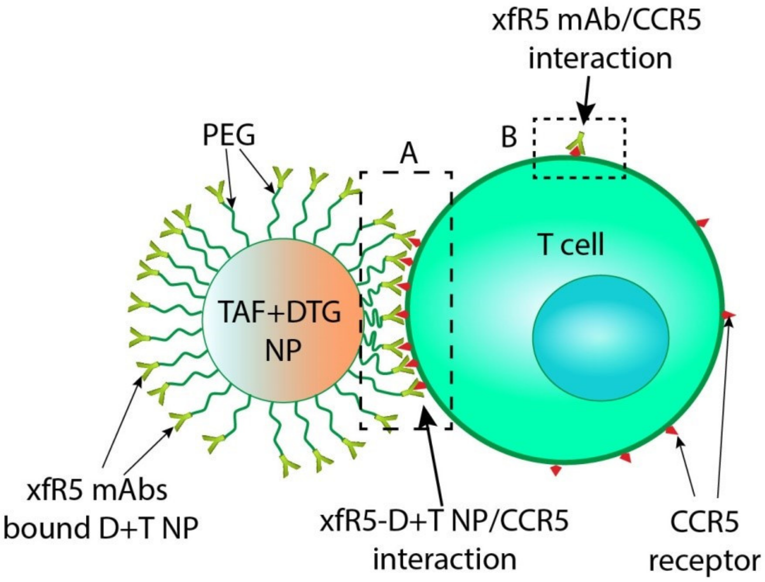

Figure S1. Schematic diagram explains the xfR5 mAb interaction with CCR5 receptors on T cellbinding. A: Multivalent binding of xfR5-D+T NP compared to B: monovalent binding of free xfR5mAb.

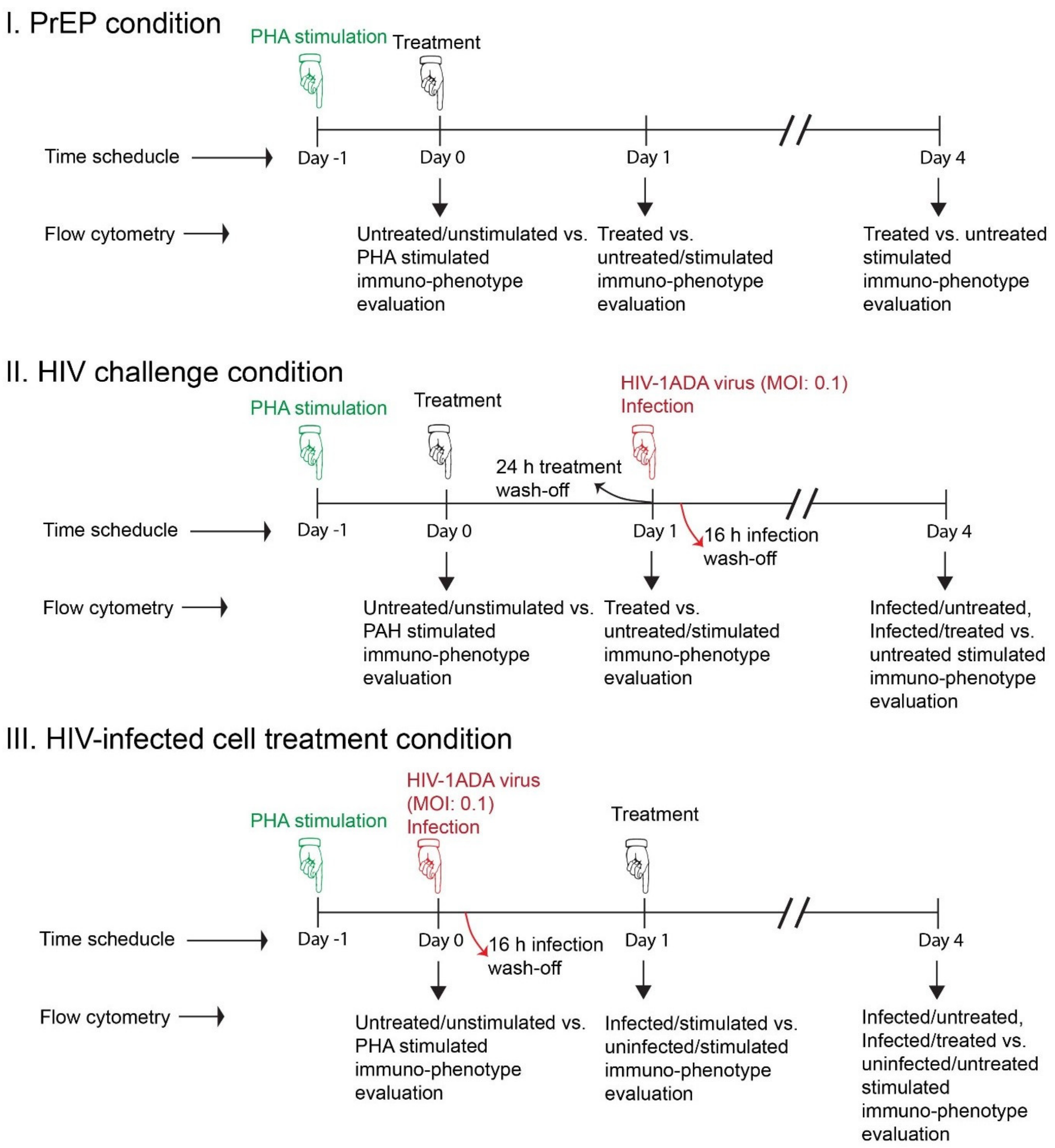

Figure S2. Schematic diagram explaining immunophenotype study design. I) Protective treatment effect on cellular immunophenotype (PrEP condition). II) HIV challenge effect on cellularimmunophenotype in presence and absence of treatment (HIV challenge condition). III) Immunological effect of treatment on HIV infected cells (HIV-infected cell treatment condition). Allcells were PHA stimulated on Day -1. A group of untreated/unstimulated cells were evaluated onDay 0 to assesses initial immune-phenotype in absence of stimulation or treatment or infection, respectively.

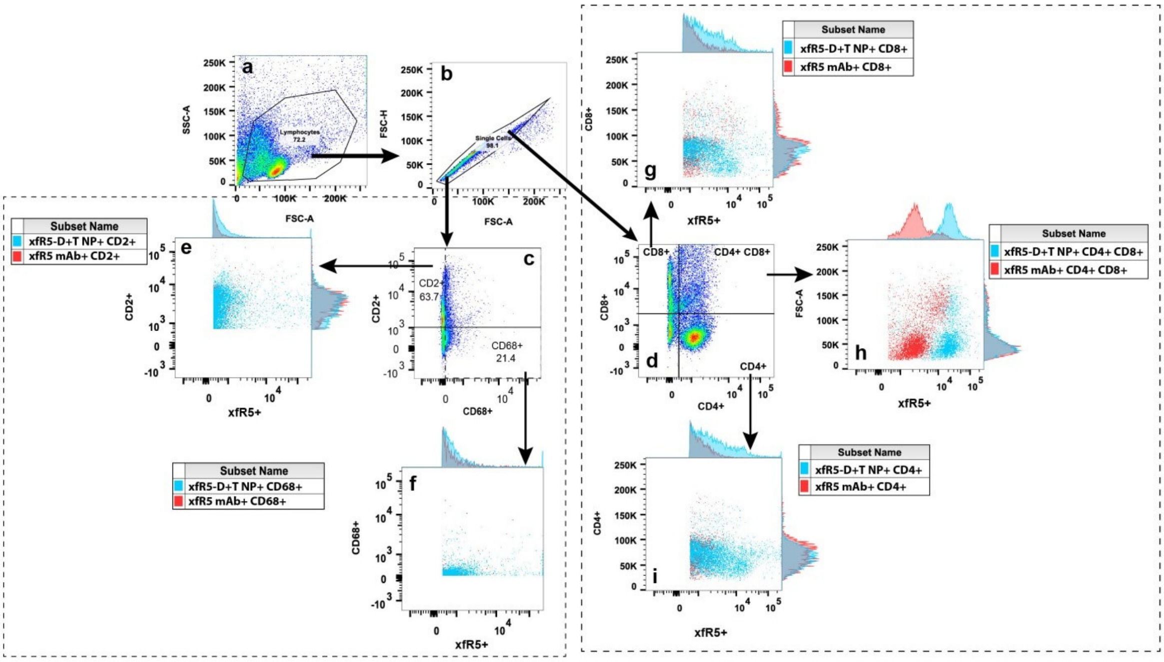

Figure S3. Flow cytometry gating strategy to evaluate T cell binding affinity. The flow cytometry plots represent the percentage (%) of the relative number of cells in the respective populations. The primary PBMCs were gated first for (**a**) lymphocytes (FSC-A vs. SSC-A), from which (**b**) singlecell (FSC-A vs. FSC-H) were gated. Among single-cell lymphocytes were further gated for (**c**) CD2+ vs. CD68+ cells and (**d**) CD4+ vs. CD8+ cells. The graphical images represent comparativebinding potency of xfR5-D+T NPs and xfR5 mAbs (free) after treatment (at 66.5 nM of xfR5 mAbconcentration as NP bound or free), with primary CD2+ (Dendritic cells or HIV latent T-cells, (**e**), CD68+ (monocytes, (**f**), CD8+ (**g**), CD8+CD4+ (**h**) and CD4+ (**i**) cell-types among PBMCspopulation. For setting the negative and positive gate, during each experiment, respective isotype controls and several channel markers were used, respectively. All samples were compensated during the sample run.

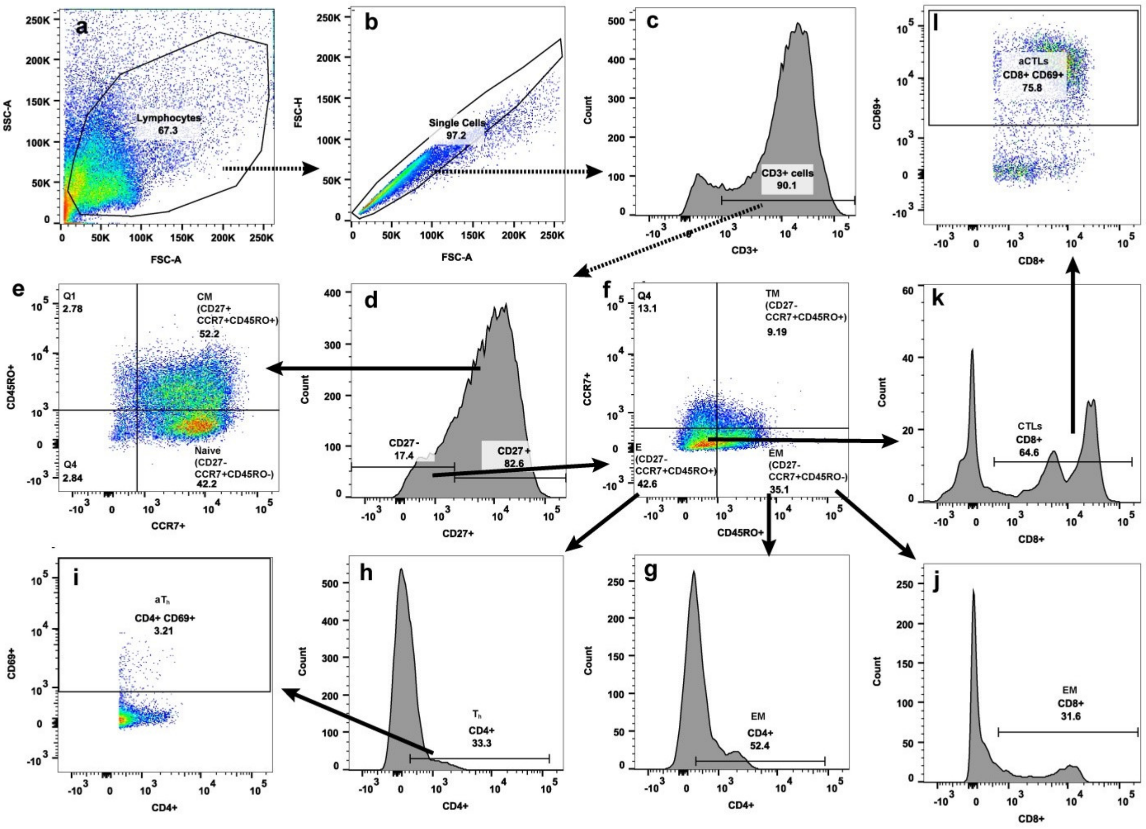

Figure S4. Flow cytometry gating strategy to evaluate T cell differentiation pattern upon xfR5- D+T NPs and xfR5 mAbs (free) treatment (at 66.5 nM of xfR5 mAb concentration as NP bound or free). The primary PBMCs were gated against T-lymphocytes (CD3), helper T-cells (CD4), Cytotoxic T-cells (CD8), Memory T-cells (CD45RO), Transition T-cells (CCR7), Activated T-cells(CD69), and Intermediate memory T-cells (CD27). The flow cytometry plots represent the relativenumber of cells in the respective populations (% mean). The gatings are, (**a**) Live cell; (**b**) Singlecells, (**c**) T lymphocytes (CD3+). The CD3+ cells were further gated as histogram for CD27+/− cells (**d**). The CD27+ gated population was then sub-gated for CD45RO+ vs CCR7+ population. (**e**) to determine CM (CCR7+ CD45RO+), and naïve (CCR7+ CD45RO−). Similarly, CD27− gated population was then sub-gated for CD45RO+ vs CCR7+ population (**f**) to determine EM (CCR7− CD45RO+), TM (CCR7+ CD45RO+) and E (CCR7− CD45RO−) sub-populations. Among EM population, % of CD4+ cells (**g**) and EM CD8+ cells (**i**) where evaluated. The E population was further sub-gated for (**h**) T helper (T_h, CD4+) and (k) cytotoxic T lymphocytes (CTLs, CD8+). Further, activated (**i**) T helper (aT_h, CD4+ CD69+) and (**l**) cytotoxic T lymphocytes (CTLs, CD8+) were estimated among T_h and CTLs population, respectively. During each experiment respective isotype controls and respective channel marker were used for setting the negative and positive gate, respectively. All samples were compensated during sample run.

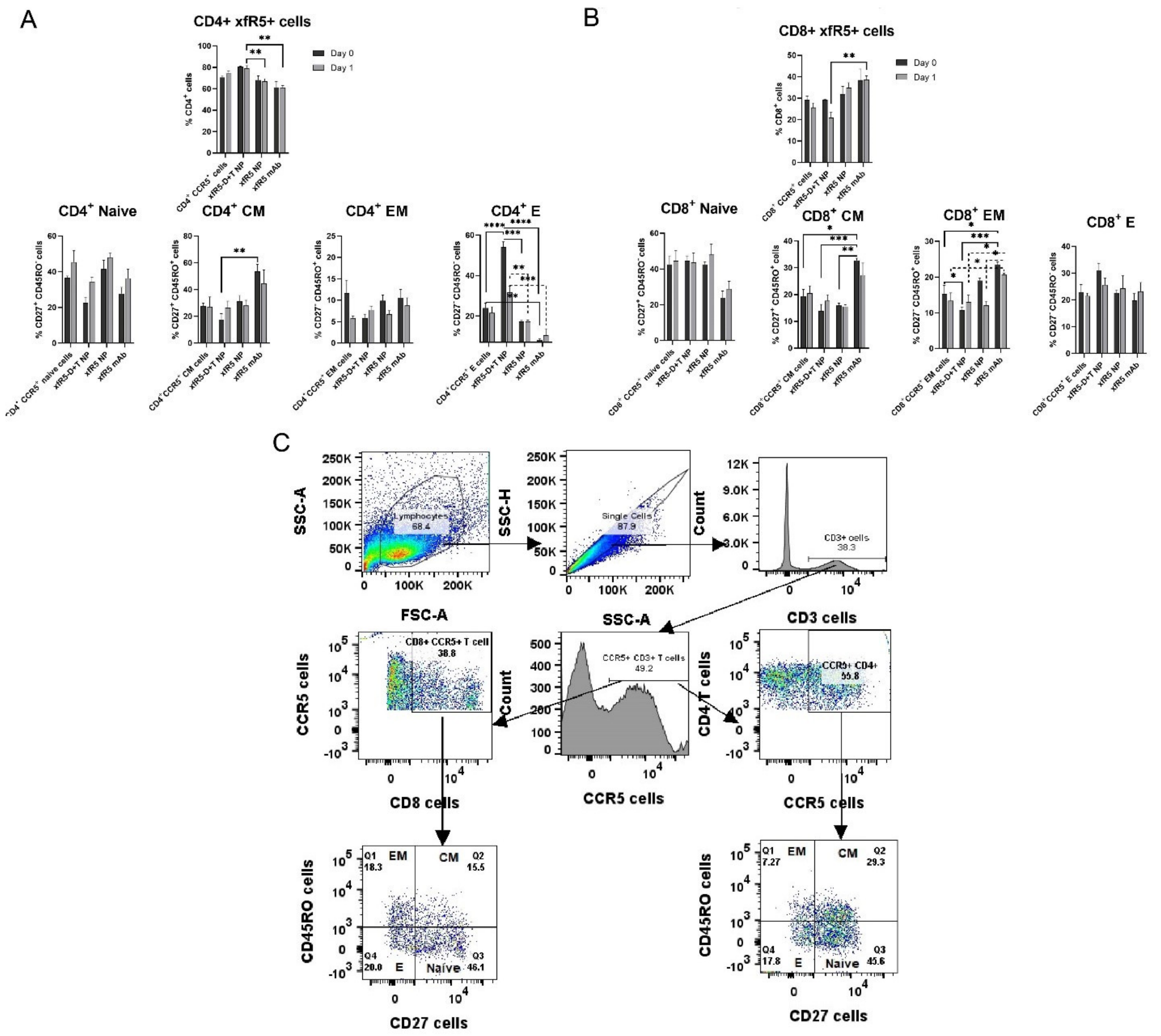

Figure S5. A graphical presentation of comparative binding of Cy3 tagged xfR5-D+T NPs vs different variables (i.e., untreated, xfR5 mAb and xfR5 NPs) with (**A**) CD4+ T cells and its sub- types; and (**B**) with CD4+ T cells and its sub-types. (**C**) Gating strategy of this immunotyping study.

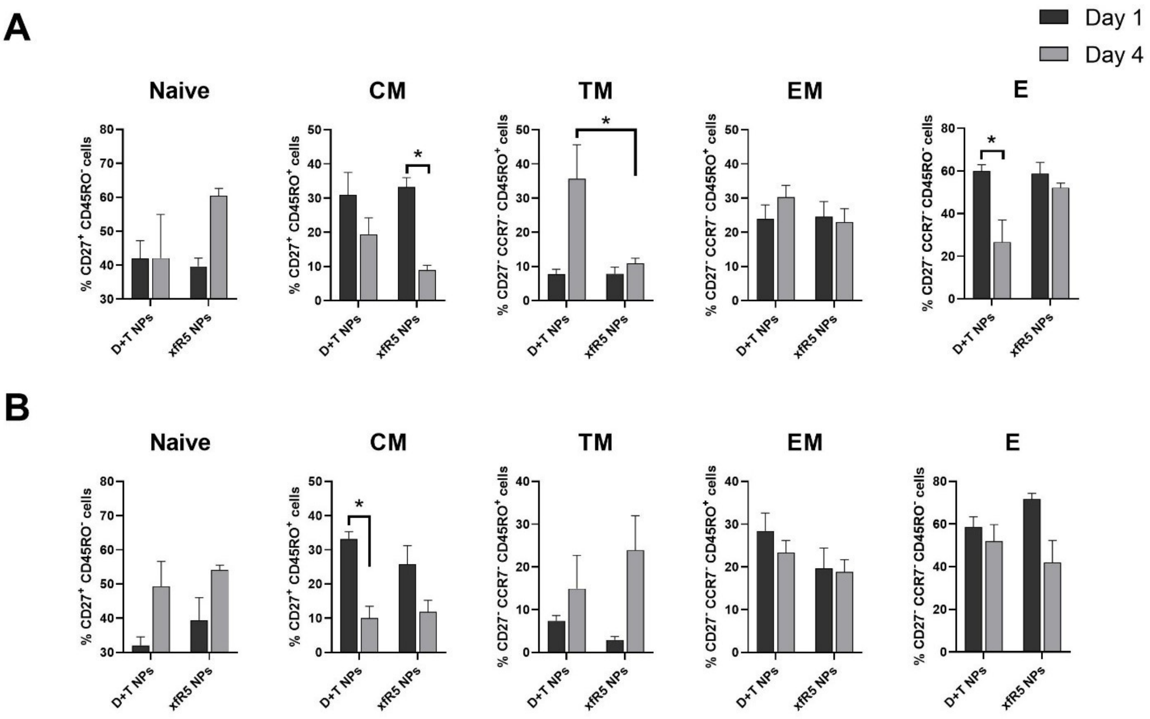

Figure S6. T-cell differentiation phenotype after D+T NP vs xfR5 NPs treatment under (**A**) protection against HIV challenge (HIV prophylaxis), and (**B**), HIV infected T-cell treatment (HIV treatment) condition. A) The immunophenotyping pattern were evaluated after cell treatment followed by $HIV\text{-}1_{ADA}$ challenge condition. B) Immunophenotyping pattern evaluation of HIV infected followed by treatment condition. The differentiation pattern was evaluated following naïve($CD27^{+}CD45RO^{-}$ cells), CM ($CD27^{+}CD45RO^{-}$ cells), TM, EM, and E sub-population as depicted in x-axis; whereas the y-axis represents respective marker % positive cells. The data presented as mean ± SEM of three independent experiments on three healthy donors (n=3). The significancewas determined by two-way ANOVA analysis followed by Tukey's multiple comparisons test andthe asterisk (*) symbol represents the significance level corresponding to *P* values <0.05.

Table S1. Comparative one-way ANOVA analysis (using Tukey's multiple comparisons test) of CC_{50} of TZM-bl and PBMCs ('ns'= non-significant 'p' value).

Cell type	Compared treatment variable	Significant?	Summary	AdjustedP Value
TZM-bl	xfCCR5 D+T NPs vs. xfCCR5 NPs	Yes	****	<0.0001
	xfCCR5 D+T NPs vs. D+T NPs	No	ns	0.6238
	xfCCR5 D+T NPs vs. xfCCR5 mAb	Yes	****	<0.0001
	xfCCR5 NPs vs. D+T NPs	Yes	****	<0.0001
	xfCCR5 NPs vs. xfCCR5 mAb	No	ns	0.0572
	D+T NPs vs. xfCCR5 mAb	Yes	****	<0.0001
PBMCs	xfCCR5 D+T NPs vs. xfCCR5 NPs	Yes	**	0.0027
	xfCCR5 D+T NPs vs. D+T NPs	No	ns	0.1649
	xfCCR5 D+T NPs vs. xfCCR5 mAb	No	ns	0.7895
	xfCCR5 NPs vs. D+T NPs	No	ns	0.0581
	xfCCR5 NPs vs. xfCCR5 mAb	Yes	**	0.0085
	D+T NPs vs. xfCCR5 mAb	No	ns	0.5218

Table S2. Comparative one-way ANOVA analysis (using Tukey's multiple comparisons test) of $IC_{50}$of TZM-bl and PBMCs ('ns'= non-significant 'p' value).

Cell type	Compared treatment variable	Significant?	Summary	AdjustedP Value
**TZM-bl**	xfCCR5 D+T NPs vs. xfCCR5 NPs	No	ns	>0.9999
	xfCCR5 D+T NPs vs. D+T NPs	No	ns	0.9998
	xfCCR5 D+T NPs vs. xfCCR5 mAb	Yes	****	<0.0001
	xfCCR5 NPs vs. D+T NPs	No	ns	0.9999
	xfCCR5 NPs vs. xfCCR5 mAb	Yes	****	<0.0001
	D+T NPs vs. xfCCR5 mAb	Yes	****	<0.0001
**PBMCs**	xfCCR5 D+T NPs vs. xfCCR5 NPs	Yes	****	<0.0001
	xfCCR5 D+T NPs vs. D+T NPs	Yes	****	<0.0001
	xfCCR5 D+T NPs vs. xfCCR5 mAb	Yes	****	<0.0001
	xfCCR5 NPs vs. D+T NPs	Yes	***	0.0004
	xfCCR5 NPs vs. xfCCR5 mAb	Yes	****	<0.0001
	D+T NPs vs. xfCCR5 mAb	Yes	****	<0.0001
